# Efficacy and safety of vernakalant for cardioversion of recent-onset atrial fibrillation: A protocol for systematic review and meta-analysis

**DOI:** 10.1097/MD.0000000000029038

**Published:** 2022-03-11

**Authors:** Hong Li, Yi Liang, Xuejing Song, Wu-Sha Liu-Huo, Wen Chen, Chao Tang, Lizhi Zhao, Xue Bai

**Affiliations:** a Southwest Medical University, Luzhou, Sichuan, China; b The Affiliated Traditional Chinese Medicine Hospital of Southwest Medical University, Luzhou, Sichuan, China.

**Keywords:** atrial fibrillation, meta-analysis, protocol, randomized controlled trials, vernakalant

## Abstract

**Background:**

Atrial fibrillation (AF) is the most common tachyarrhythmia encountered in clinical practice and is associated with substantial morbidity and mortality. This study aimed to determine the efficacy and safety of vernakalant for cardioversion of recent-onset AF.

**Methods:**

A comprehensive systematic literature search will be conducted in Cochrane Library, PubMed, Web of Science, EMBASE, for randomized controlled trials (RCTs) about the vernakalant with AF. Two reviewers will independently assess the quality of the selected studies according to the Cochrane Collaboration's tool for RCTs. The bias risk of the RCT will be assessed by the Cochrane risk of bias (ROB) tool. The quality of the evidence will be evaluated by Grading of Recommendations Assessment Development and Evaluation (GRADE) system. Results from these questions will be graphed and assessed using Review Manager 5.3.

**Results:**

The results of this meta-analysis will be published in a peer-reviewed journal.

**Conclusion:**

This review will evaluate the safety and efficacy of vernakalant for patients with AF, provide more recommendations for patients or researchers, and high-level evidence for clinical decision-making.

## 1 Introduction

Atrial fibrillation (AF) is the most common tachyarrhythmia encountered in clinical practice and is associated with substantial morbidity and mortality, characterized by irregularly irregular atrial electrical activity.^[1]^ Electrocardiographic features of AF include a rapid irregular fibrillatory wave and disappearance of the P-wave.^[2]^ In the past decades, lots of hypotheses of AF have been suggested, however, the exact pathogenesis is still remains elusive. Now, most scholars agree that the atrial trigger mechanism and maintenance mechanism are the main mechanisms for the occurrence and development of AF.^[3–5]^ AF can cause rapid ventricular rates resulting in hemodynamic deterioration. and reduction of cardiac output.^[6]^ A study has shown that the incidence of stroke and vascular embolism in patients with AF is 7 times that in patients without AF, which has become one of the major human disability factors in the world.^[7]^ A large-scale systematic epidemiological study showed that the incidence, morbidity, disability and mortality of AF in 2010 were significantly higher than in 1990.^[8]^ Treatment of AF is generally subdivided into medication, cardioversion, ablation, and surgical treatment. The main treatment methods are rhythm control, heart rate control, and anticoagulation therapy.^[9]^ Current guidelines recommend that rhythm control as the preferred treatment for AF.^[10]^ The overall electrical cardioversion success rate is 88%. However, sinus rhythm cannot be sustained for long after cardioversion,^[11]^ and often requires effective supplemental rhythm control treatment. As a result, more clinical centers are choosing drug cardioversion. Because the patients with AF (within 7 days) do not have an effective maintenance mechanism in their atria, which allows us to restore the sinus rhythm of patients with AF.

As a new antiarrhythmic drug, vernakalant has been generally considered as selectively blocking atrial-specific ultra-rapid delayed rectifier K+ current (IKur) and acetylcholine-sensitive current (IKAch).^[12]^ At the same time, vernakalant increases the effective refractory period of the atrial muscle, leading to rapid cardioversion of AF by specific blocking of atrial-specific frequency-dependent sodium channel.^[12]^ Since vernakalant has no obvious selectivity for ventricular muscles, it will not cause significant QT interval prolongation, which can avoid affecting the transmural dispersion of repolarization (TDR), and reduce the incidence of fatal ventricular arrhythmias. A fundamental mechanism study found that vernakalant can reduce intracellular calcium overload after blocking the delayed sodium channel current (INa) and inhibit delayed afterdepolarizations (DADs), avoiding the formation of atrial muscle trigger mechanism, which can maintain sinus rhythm effectively.^[13]^ Therefore, we intend to conduct a systematic review and meta-analysis to clarify the efficacy and safety of vernakalant for cardioversion of recent-onset AF.

## 2 Materials and methods

### 2.1 Study registration

This protocol will be reported according to preferred reporting items for systematic review and meta-analysis protocols (PRISMA-P). At the same time, the protocol for this systematic review and meta-analysis has been registered in PROSPERO. Registration number CRD42022300989 (URL: https://www.crd.york.ac.uk/prospero/display_record.php?ID=CRD42022300989).

### 2.2 Search strategy

We will search PubMed, The Cochrane Library, EMBASE, and Web of Science with no date or language restrictions. The search string will be built as follows: (atrial fibrillation OR AF AND vernakalant). The electronic database search will be supplemented by a manual search of the reference lists of included articles.

### 2.3 Inclusion and exclusion criteria

#### 2.3.1 Types of studies

Randomized controlled trials (RCTs) comparing vernakalant with antiarrhythmic drugs or active intervention in patients with recent-onset AF will be included without language restriction.

#### 2.3.2 Types of participants

Patients with AF of onset ≤7 days (as diagnosed by a clinician, or using any recognized diagnostic criteria^[14]^) will be included.

#### 2.3.3 Types of interventions

The intervention was intravenous vernakalant administration as a first-line agent with the purpose of cardioversion compared with placebo or other antiarrhythmic drugs.

#### 2.3.4 Types of outcome measures

Main outcomes:

1. 90-minute cardioversion success rate
2. sustained sinus rhythm at 24 hours

Additional outcome:

1. Adverse reaction rate
2. Recurrence of AF

### 2.4 Exclusion criteria

The following criteria will be excluded:

1. the study included too little information or data could not be obtained, such as review articles, editorials, comments, and protocols;
2. conference abstracts, and duplicate reports of the same study.

### 2.5 Study selection

Data were extracted and cross-checked for accuracy independently by 2 investigators using a self-designed data form. Discrepancies were resolved by consensus or a third investigator.

### 2.6 Data extraction

For each study, the following data were extracted: year of publication, comparator drug, sample size, proportion of patients with structural heart disease (including coronary heart disease, ischemic heart disease, valvular heart disease, congestive heart failure, and cardiomyopathy), study design, definition of recent-onset AF, cardioversion rate within 90 minutes, median time of cardioversion, and proportion of patients without symptoms at 90 minutes. Safety outcomes from RCTs including treatment-emergent adverse events within 24 hours after dose, SAEs, and adverse events leading to drug discontinuation which related to vernakalant were also extracted.

### 2.7 Assessment of risk of bias in included studies

Two reviewers will independently assess the quality of the selected studies according to the Cochrane Collaboration's tool^[15]^ for RCTs. Items will be evaluated in three categories: low risk of bias (ROB), unclear bias and high ROB. The following characteristics will be evaluated: random sequence generation (selection bias), allocation concealment (selection bias), blending of participants and personnel (performance bias), incomplete outcome data (attrition bias), selective reporting (reporting bias), other biases. Results from these questions will be graphed and assessed using Review Manager 5.3.

### 2.8 Data synthesis and analysis

Risk ratio (RR) for both fixed and random effects models (weighting by inverse of the variance) will be used. A continuity correction will also be used for cells with zero values. Between-study heterogeneity will be assessed using the τ^2^, χ^2^ (Cochran Q) and I^2^ statistics. According to the Cochrane handbook, the I^2^ will be considered non-important (<30%), moderate (30%–60%), and substantial (>60%). Results will be assessed using forest plots and presented as RRs for the main outcome and secondary outcomes. An influence analysis will be performed to ascertain the results of the meta-analysis by excluding each of the individual studies. Publication bias will be assessed by a funnel plot for meta-analysis and quantified by the Egger method. Statistical analysis will be conducted using STATA software for Mac v15.0 (Stata Corp., College Station, TX) [module “meta”] and R studio v1.0.136 (The R Foundation for Statistical Computing) [package “meta v4.2”].

### 2.9 Subgroup analysis and investigations of heterogeneity

Pre-specified subgroup analyses were performed hypothesizing that vernakalant would be superior in the following subgroups:

1. patients treated with placebo as compared to an active drug,
2. patients who were in the post-operative period following cardiac surgery compared to those who were not,
3. patients without prior AF as compared to those with a history of AF,
4. patients without a history of congestive heart failure as compared to those with a history of congestive heart failure,
5. patients without as compared to those with a history of ischemic heart disease.

### 2.10 Sensitivity analyses

We plan to conduct a sensitivity analysis^[16]^ and limit the analysis to studies where we consider the ROB to be low or unclear.

### 2.11 Certainty assessment

Two trained GRADE^[17]^ methodologists will use Grades of Recommendation, Assessment, Development, and Evaluation (GRADE) system to assess the certainty (quality) of evidence associated with specific outcomes and constructed a summary of findings table. Assessment of the quality of evidence considers study methodological quality, directness of the evidence, heterogeneity of data, precision of effect estimates, and risk of publication bias. For each significant outcome, we will be awarded 4 points to begin with as these were based on randomized trials and assessed the limitations that can reduce the quality of this evidence.

### 2.12 Ethics and dissemination

It is not necessary for ethical approval because it is based on published studies. The protocol will be disseminated in a peer-reviewed journal or presented at a topic-related conference.

## 3 Discussion

This review will evaluate the safety and efficacy of vernakalant for patients with AF. Vernakalant has been generally considered as selectively blocking atrial-specific IKur and IKAch rapid cardioversion of AF.^[12]^ Considering the importance of maintaining sinus rhythm in the treatment of AF, we hope this systematic review can provide the health care workers with more high-quality evidence to choose vernakalant for patients with recent-onset AF.

## Author contributions

**Conceptualization:** Hong Li, Yi Liang.

**Data curation:** Xuejing Song, Wu-Sha Liu-Huo

**Methodology:** Wen Chen, Chao Tang

**Project administration:** Xue Bai, Lizhi Zhao

**Writing – original draft:** Hong Li, Yi Liang.

**Writing– review & editing:** Hong Li, Xuejing Song.

## Correction

This study was supported by the Project of Science and Technology Department of Sichuan Province (2020YJ0437), Luzhou Science and Technology Project (2020-SYF-30), and Sichuan Administration of Traditional Chinese Medicine ( No. 2021MS114).
